# Pediatric Airway Stent Designed to Facilitate Mucus Transport and Atraumatic Removal

**DOI:** 10.1109/TBME.2019.2910551

**Published:** 2019-04-11

**Authors:** Junhyoung Ha, Abhijit Mondal, Zhanyue Zhao, Aditya K. Kaza, Pierre E. Dupont

**Affiliations:** 1 Department of Cardiovascular SurgeryBoston Children's Hospital, Harvard Medical School; 2 Department of Cardiovascular SurgeryBoston Children's Hospital, Harvard Medical SchoolBostonMA02115USA

**Keywords:** Tracheobronchomalacia, airway stent

## Abstract

*Objective:* The goal was to develop a pediatric airway stent for treating tracheobronchomalacia that could be used as an alternative to positive pressure ventilation. The design goals were for the stent to allow mucus flow and to resist migration inside the airways, while also enabling easy insertion and removal. *Methods:* A helical stent design, together with insertion and removal tools, is presented. A mechanics model of stent compression is derived to assist in selecting stent design parameters (pitch and wire diameter) that provide the desired amount of tracheal support, while introducing the minimal amount of foreign material into the airway. Worst-case airway area reduction with stent support is investigated experimentally using a pressurized tracheal phantom matched to porcine tracheal tissue properties. The stent design is then evaluated in a porcine *in vivo* experiment. *Results:* Phantom testing validated the mechanics model of stent compression. *In vivo* testing demonstrated that the stent was well tolerated by the animal. Since the helical design covers only a small portion of the epithelium, mucus transport through the stented region was minimally impeded. Furthermore, the screw-like stent resisted migration, while also providing for atraumatic removal through the use of an unscrewing motion during removal. *Conclusion:* The proposed stent design and tools represent a promising approach to prevent airway collapse in children with tracheobronchomalacia. *Significance:* The proposed technology overcomes the limitations of existing airway stents and may provide an alternative to maintaining children on a ventilator.

## I. Introduction

Tracheobronchomalacia is the most common congenital defect of the central airways [1] and has been identified in up to 15% of infants and 30% of young children undergoing bronchoscopic examination for respiratory distress [2]. The condition arises due to intrinsic weakness of the wall and cartilaginous support. For these children, during dynamic expiration and coughing at low lung volume, pleural pressure exceeds intraluminal pressure resulting in airway collapse [3], [4]. The standard treatment is positive pressure ventilation of 5–10 cm H$_2$O [5], which raises the intraluminal pressure sufficiently to prevent collapse during expiration [5]–[7]. During the 3–9 month treatment period, however, the child must remain connected to a ventilator and close monitoring is needed to provide regular suctioning of the endotracheal tube. Even with suctioning, the inability to clear mucus leads to an increased risk of airway infections including pneumonia and tracheitis [8]–[10].

**Fig. 1.**
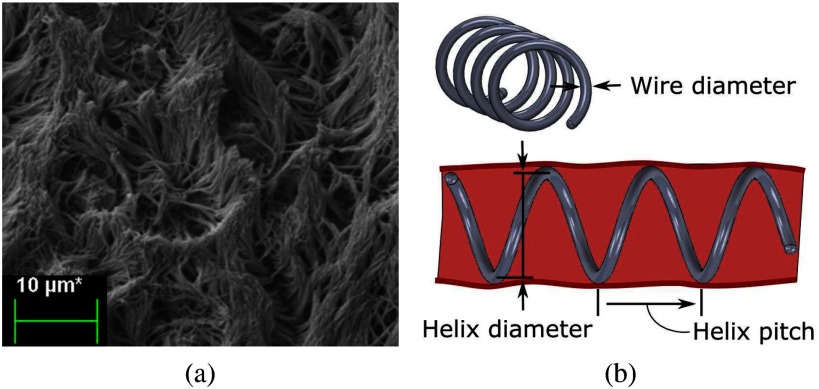
Tracheal cilia and mucus transport. (a) Scanning Electron Microscopic (SEM) image of cilia on porcine tracheal epithilium. Beating motion of cilia transports mucus and debris out of the airway. (b) Helical stent design parameters.

An alternative approach to positive pressure ventilation is to employ a physical stent to prevent airway collapse. Since existing airway stents are sized for adults, some neonates have been treated with metal mesh vascular stents [11]. Similar to the experience with metal airway stents in adults, however, these stents induce the growth of granulation tissue through the mesh requiring a very invasive removal procedure and, furthermore, can erode through the airway and into adjacent structures [12], [13]. To avoid these issues, solid silicone tubes have been developed for the adult population, but their thickness reduces the airway diameter and they have a high migration rate [14], [15]. They also block mucociliary function (Fig. 1(a)) over the length of the stent resulting in mucous plugging and inspissated secretions that impede gas exchange [12], [16].

Given the substantial promise of resorbable stents to treat luminal disease, resorbable airway stents are being developed [17]. Current resorbable designs, however, tend to fragment during resorption and these fragments can migrate to and block the distal airways. Consequently, weekly bronchoscopies are recommended to monitor the stent [18]–[20]. Recently, surgically-placed external resorbable tracheal splints have been developed which show great promise for those patients with malacic airways who also require thoracic surgery [21]. For those patients who are not undergoing such surgeries, however, an internally-placed stent that avoids the existing shortcomings is sorely needed.

A stent design appropriate for neonates and infants should address four criteria. The design should:

1) minimally impede cilia-mediated mucus streaming,
2) minimize stent migration,
3) enable atraumatic removal, and
4) minimize the amount of foreign material in the airways.

The contribution of this paper is to develop a helical stent, Fig. 1(b), addressing these criteria as well as tools for its delivery and removal. Furthermore, we validate the design through bench and in vivo experiments. The remainder of this paper is arranged as follows. In the next section, we propose a helical stent design and derive a mechanics-based model for computing stent deformation under a given external pressure. Next, we develop a silicone trachea phantom to evaluate the effect of tissue deformation on airway area reduction and the relationship with stent pitch and wire diameter. Next, stent delivery and removal tools are described, followed by in vivo stent safety validation in a porcine model. Conclusions are presented in the final section.

## II. Stent Design

Two airway mucus flow patterns have been observed in humans. The first favors linear transport along the posterior tracheal wall [22] while the second follows a spiral pattern [22], [23]. Ideally, a stent should not obstruct this flow. A helical stent geometry of the appropriate chirality and pitch will not impede mucus flow for those with a spiral pattern. Furthermore, analogous to a screw, this geometry will resist migration while also enabling atraumatic removal in the case of endothelialization via an unscrewing motion. While a helical stent will interrupt mucus flow for those with linear transport patterns, the width of the interruption will be small as long as the wire diameter used to construct the stent is small.

The design of a helical stent involves selecting the wire diameter large enough and the helical pitch small enough to provide radial support equivalent to positive pressure ventilation. At the same time, the minimum wire diameter and maximum pitch satisfying the equivalent support criterion should be used in order to minimize interference with mucus streaming while also introducing the minimum amount of foreign material into the airways.

**Fig. 2.**
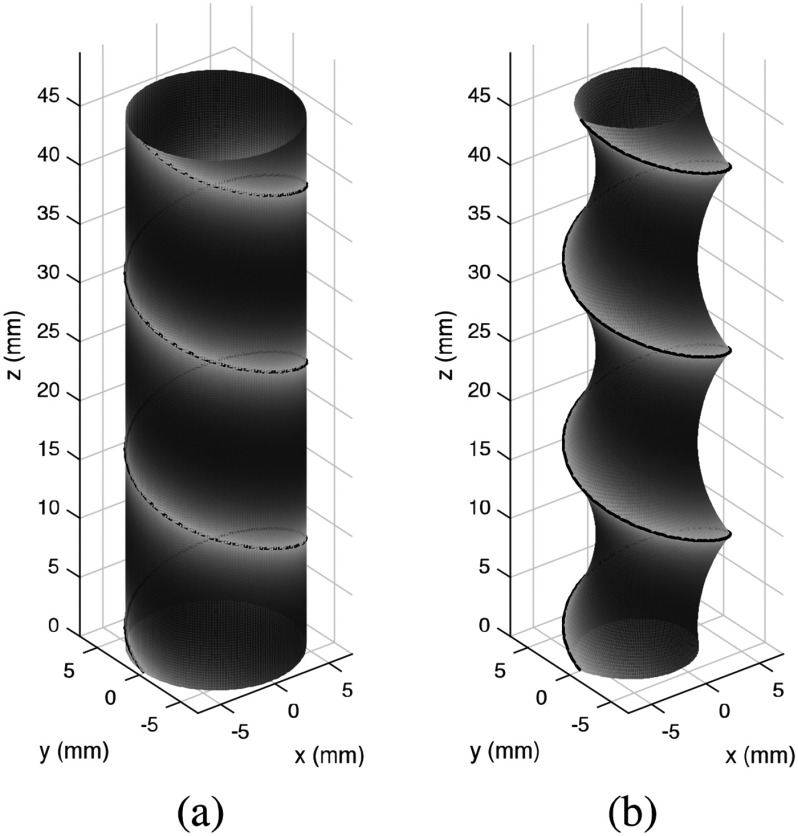
Idealized deformation of stented trachea under external pressure. (a) Unloaded stent and trachea. (b) Under pressure, stent diameter decreases and tracheal tissue is stretched inward.

In other words, we wish to solve for the pairs of minimum wire diameter and maximum pitch for which the airway cross-sectional area is reduced by an acceptable value when the trachea experiences an external pressure equivalent to positive pressure ventilation. To solve this problem, we need to estimate the reduction in stented airway cross sectional area in response to an external pressure, as idealized in Fig. 2.

In this idealized model, we assume a worst-case scenario in which the airway is comprised of a uniform tissue without reinforcing cartilage rings. The reduction in cross sectional area can be attributed to two components. First, the external pressure causes the stent to reduce in diameter. Second, the tissue between helical coils stretches to form an asymmetrical hourglass shape. To solve this design problem, we need to be able to estimate both of these phenomena. In this section, we derive an analytical model for stent deformation. In the following section, we experimentally validate this model while also experimentally estimating area reduction due to tissue deformation.

### A. Stent Diameter as a Function of Uniform External Pressure

**Fig. 3.**
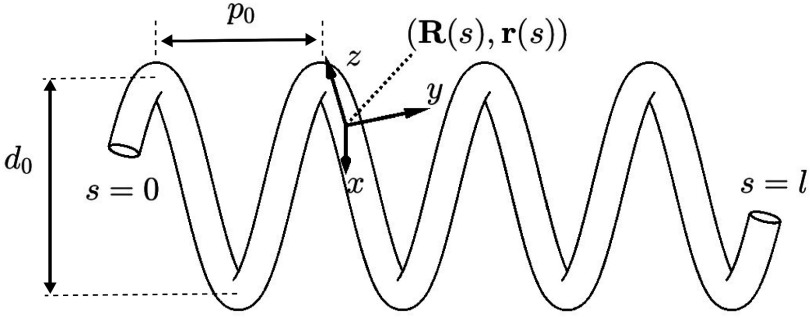
Parameters of helical stent model. $\mathbf {R}(s)$ and $\mathbf {p}(s)$ define the body frame and wire centerline, respectively, as functions of arc length $s$.

Consider the helical stent shown in Fig. 3, whose unloaded pitch and helix diameter are $p_0$ and $d_0$, respectively. While beyond the scope of this paper, it can be shown that when a helix comprised of a linearly elastic material is compressed radially, its central portion (away from the ends) remains helical, but its pitch and length increase [24], [25]. While the ends deviate from a helical shape, stents are sized such that they extend on both ends beyond the portion of the airway that requires support. Consequently, we only consider the central helical portion in our model below.

**Fig. 4.**
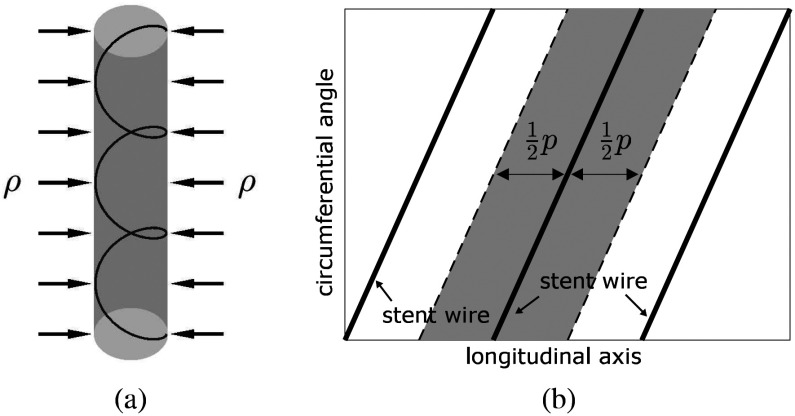
Relationship between external pressure and force per unit length on helical wire. (a) Uniform pressure, $\rho$, applied to cylindrical trachea supported by a helical stent. (b) Cylindrical coordinate graph showing area under pressure supported by single coil.

The derivation proceeds in two steps. Given an initial helical elastic stent, we first derive a method to solve for the pitch, $p$, associated with a reduced diameter, $d < d_0$. Next, we solve for the pressure associated with a reduced diameter under the assumption that the pressure, $\mathrm{\rho }$, is distributed uniformly and can be converted into a radially directed force per unit length, $f$, as shown in Fig. 4. The conversion to $f$ is given by dividing the pressure integration in the shaded area of Fig. 4(b) by the length of the stent coil that supports the pressure:

\begin{equation*}
f = \frac{\mathrm{\rho } \pi d p }{\sqrt{p^2 + \pi ^2 d^2}} \tag{1}
\end{equation*}

#### 1) Pitch Versus Diameter

The stent wire is parameterized with an arc length parameter $s \in [0,l]$ where $l$ is the total length of the wire. Let $\mathbf {r}(s) \in \mathbb {R}^3$ denote the central line of the wire and $\mathbf {R}(s) \in SO(3)$ denote a body frame attached at $s$ where $SO(3)$ is the special orthogonal group, i.e., $\mathbf {R}(s)$ is a $3 \times 3$ matrix sastifying $\mathbf {R}(s)^T \mathbf {R}(s) = \mathbf {I}_{3 \times 3}$ and $\det (\mathbf {R}(s)) = 1$. We define the body frame $\mathbf {R}(s)$ to have its $z$-axis tangent to the wire and its $x$-axis point to the center of the helix. The differential equations for $\mathbf {R}(s)$ and ${\mathbf {r}}(s)$ are then given by

\begin{align*}
\dot{\mathbf {R}}(s) &= \mathbf {R}(s) \left[\hat{\mathbf {u}}\right]\nonumber\\
\dot{\mathbf {r}}(s) &= \mathbf {R}(s) \mathbf {e}_z \tag{2}
\end{align*}

where $\mathbf {e}_z= [0 \ 0 \ 1]^T$ and the upper dot represents the derivative with respect to $s$. The notation $[\cdot ]$ is the $3 \times 3$ skew symmetric matrix representation of 3-dimensional vector and the curvature vector $\hat{\mathbf {u}} \in \mathbb {R}^3$ is given by

\begin{equation*}
\hat{\mathbf {u}} = \left[\begin{array}{ccc}\hat{u}_x & \hat{u}_y & \hat{u}_z \end{array} \right]^T = \left[\begin{array}{ccc}0 & \displaystyle\frac{2 \pi ^2 d_0}{c_0^2} & \displaystyle\frac{2 \pi p_0}{c_0^2} \end{array} \right]^T \tag{3}
\end{equation*}

where $c_0 = \sqrt{\pi ^2 d_0^2 + p_0^2}$ is the length of a single coil.

Now consider that the helix has a reduced diameter $d (< d_0)$. Assuming the reduced shape is also a helix, its new parameters can be obtained through potential energy minimization. Let $p (> 0)$ denote the new helix pitch and $c$ and $\mathbf {u} \in \mathbb {R}^{3}$ denote the corresponding coil length and curvature vector, respectively, given by

\begin{align*}
c &= \sqrt{\pi ^2 d^2 + p^2}, \tag{4}\\
\mathbf {u} &= \left[\begin{array}{ccc}u_x & u_y & u_z \end{array} \right]^T = \left[\begin{array}{ccc}0 &\displaystyle \frac{2 \pi ^2 d}{c^2} & \displaystyle\frac{2 \pi p}{c^2}. \end{array} \right]^T \tag{5}
\end{align*}

The change in elastic potential energy is given by

\begin{align*}
E &= \frac{1}{2} \int _0^l (\mathbf {u} - \hat{\mathbf {u}})^T \mathbf {K} (\mathbf {u} - \hat{\mathbf {u}}) ds \nonumber\\
&= \frac{l}{2} (\mathbf {u} - \hat{\mathbf {u}})^T \mathbf {K} (\mathbf {u} - \hat{\mathbf {u}}) \tag{6}
\end{align*}

where $\mathbf {K} \in \mathbb {R}^{3 \times 3}$ is a stiffness matrix whose diagonal components consist of the bending stiffness $k_b$ and the torsional stiffness $k_t$, i.e.,

\begin{equation*}
\mathbf {K} = \left[ \begin{array}{ccc}k_b & 0 & 0 \\
0 & k_b & 0 \\
0 & 0 & k_t \end{array} \right]. \tag{7}
\end{equation*}

Since $d$ is given, the only unknown in the energy function (6) is $p$ as $l, \hat{\mathbf {u}}$ and $\mathbf {K}$ are known and $\mathbf {u}$ is a function of $d$ and $p$. Substituting (4) and (5) into (6), the first-order necessary condition for minimization of $E$ is

\begin{equation*}
\begin{split} \frac{dE}{dp} &= \frac{2\pi l}{c^6} \left(a_0 + a_1 p + a_2 p^2 + a_3 p^3 + a_4 p^4 \right) = 0 \end{split} \tag{8}
\end{equation*}

where

\begin{align*}
a_0 &= -k_t \hat{u}_z \pi ^4 d^4, \quad a_1 = 2\pi ^3 d^2 (k_t - 2 k_b + k_b \hat{u}_y d),\nonumber\\
a_2 &= 0, \quad a_3 = 2\pi (k_b \hat{u}_y d - k_t), \quad a_4 = k_t\mathrm{ \hat{u}_z}. \tag{9}
\end{align*}

Since $2\pi l/c^6$ is positive, the forth-order polynomial in (8) should be zero. The energy-minimizing pitch is one of the four roots of the polynomial, which can be efficiently computed as an eigenvalue problem. The pitch can be selected as the positive real root that minimizes the energy function, $E$.

#### 2) Diameter Versus Pressure

We now want to derive a relationship between the reduced helix diameter, $d$, and an externally applied pressure, $\mathrm{\rho }$ (Fig. 4). The relationship between pressure and force per unit length on the wire is given by (1). Defining $\mathbf {f} \in \mathbb {R}^3$ as the distributed force expressed in the body frame of the helix, the curvature of the reduced diameter helix, $\mathbf {u}$, must satisfy the following differential equation from Cosserat rod theory [26], [27]:

\begin{equation*}
\left[ \begin{array}{c}\dot{\mathbf {m}} \\
\dot{\mathbf {n}} \end{array} \right] = -\left[ \begin{array}{c}\mathbf {0}_{3 \times 1} \\
\mathbf {f} \end{array} \right] - \left[ \begin{array}{cc}[\mathbf {u}] & [\hat{\mathbf {e}}_z] \\
\mathbf {0}_{3 \times 3} & [\mathbf {u}] \end{array} \right] \left[ \begin{array}{c}\mathbf {m} \\
\mathbf {n} \end{array} \right] \tag{10}
\end{equation*}

Here, $\mathbf {m} \in \mathbb {R}^3$ and $\mathbf {n} \in \mathbb {R}^3$ are the force and moment acting on the helix cross section, respectively. The dot denotes the derivative with respect to arc length, $s$. Since $\mathbf {m}$ is a constant vector given by $\mathbf {m} = [0 \ m_y \ m_z]^T = \mathbf {K} (\mathbf {u} - \hat{\mathbf {u}})$, the upper part of (10) reduces to

\begin{equation*}
\mathbf {0}_{3 \times 1} = \left[ \begin{array}{c}-u_z m_y + u_y m_z -n_y \\
n_x \\
0 \end{array} \right] \tag{11}
\end{equation*}

where $n_y$ and $n_z$ are $y$ and $z$ components of $n$. Given $n_x = 0$ and $n_y = -u_z m_y + u_y m_z$ by (11), the lower part of (10) simplifies to

\begin{equation*}
\left[ \begin{array}{c}0 \\
0 \\
\dot{n}_z \end{array} \right] = -\left[ \begin{array}{c}f_x \\
f_y \\
f_z \end{array} \right] - \left[ \begin{array}{c}-u_z n_y + u_y n_z \\
0 \\
0 \end{array} \right]. \tag{12}
\end{equation*}

Neglecting friction between trachea and the stent, the $z$ component of the contact force, $f_z$, is zero as the $z$ direction of $\mathbf {R}(s)$ is always perpendicular to the contact force. Then $f_x = u_z n_y - u_y n_z$, $f_y = 0$ and $n_z$ is constant with respect to arc length. Since all the contact forces on the helix are perpendicular to the central axis of the helix, the force $n$, which is an integration of the contact forces, should also lie on a plane perpendicular to the helix central axis. Then $n_z$ is dependent on $n_y$ and the helix angle $\theta$:

\begin{equation*}
n_z = -n_y \cot \theta . \tag{13}
\end{equation*}

Substituting $\cot \theta = \frac{\pi d}{p}$ and (5) into (13) yields

\begin{equation*}
n_z = -n_y \frac{u_y}{u_z}. \tag{14}
\end{equation*}

Combining (11), (12) and (14), the contact force $f$ is given by

\begin{equation*}
\mathbf {f} = \left[ \begin{array}{ccc}f_x & 0 & 0 \end{array} \right]^T \tag{15}
\end{equation*}

where

\begin{equation*}
f_x = \frac{u_y^2 + u_z^2}{u_z} \left((k_t - \mathrm{k_b)} u_y u_z - k_t \hat{u}_z u_y + k_b \hat{u}_y u_z \right). \tag{16}
\end{equation*}

Recalling (1), the distributed force relates to pressure by

\begin{equation*}
f = \frac{\mathrm{\rho } \pi d p }{\sqrt{p^2 + \pi ^2 d^2}} \tag{17}
\end{equation*}

The reduced diameter $d$ can be computed by equating the two force equations (16) and (17). This gives a root finding problem, which can be solved by a standard method, e.g., Newton-Raphson, with the initial guess given by $d = d_0$.

This model enables us to estimate the changes in diameter and pitch associated with an externally applied pressure. We also need to be able to estimate the reduction in cross section area due to stretching of the tracheal tissue. This is performed experimentally using a phantom model as described below.

## III. Estimating Tracheal Airway Reduction in a Phantom Model

We conducted experiments in which we subjected stented phantom trachea to 10 cm H$_2$O pressure and measured the reduction in cross sectional area of both the stent and the tracheal phantom. (Recall Fig. 2.) The goal was to solve for the pairs of minimum wire diameter and maximum pitch for which airway area is reduced by an acceptable value under tracheal pressure equivalent to positive pressure ventilation. To match the airway of the 20 kg pig used in our in vivo experiment, we consider stenting a 10 cm length of 12 mm diameter trachea. In order to avoid stent migration, the stent outer diameter is chosen to be 2 mm greater than the tracheal diameter.

### A. Stent Fabrication

The stents were fabricated from NiTi wire for its biocompatibility and superelasticity. The set of stent parameters to be tested was selected as follows. To minimize the effect on mucus transport, we first selected two readily-available NiTi wire diameters less than 1 mm (0.38 and 0.51 mm). The model of Section II-A was then used to solve for the practical range of pitches to be considered. To solve for the maximum pitch, we computed the pitch for the smaller wire diameter that would result in a 30% reduction in cross sectional area under 10 cm H$_2$O pressure. We selected the minimum pitch as that which resulted in a difference in cross sectional area of less than 5% for the two wire diameters under the same radial pressure. This range was used since larger pitches would result in too much airway collapse while smaller pitches, which are less desirable for mucus transport, would produce similar results for the two wire diameters.

The minimum and maximum pitches obtained in this way corresponded closely to helix angles of $\text{15}^\circ$ and $\text{35}^\circ$, respectively. Consequently, we designed stents with helix angles of $\lbrace \text{15}^\circ, \text{20}^\circ, \text{25}^\circ, \text{30}^\circ, \text{35}^\circ \rbrace$. This resulted in 10 stents with the following parameters.

\begin{align*}
d_{\text{wire}} \in &\lbrace 0.38\text{ mm}, 0.51\text{ mm}\rbrace, \tag{18}\\
d_0 &= 12.7\text{ mm} + d_{\text{wire}}, \tag{19}\\
p_0 \in &\lbrace 10.7\text{ mm}, 14.5\text{ mm}, 18.6\text{ mm}, 23.0\text{ mm}, 27.9\text{ mm}\rbrace . \tag{20}
\end{align*}

**Fig. 5.**
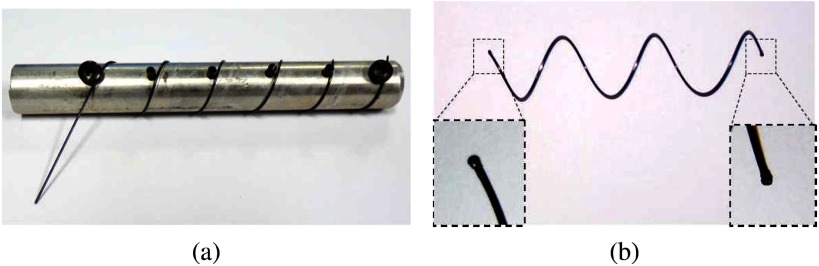
Stent fabrication. (a) NiTi wire is wound and fixed on cylindrical template of desired diameter. (b) Stent with magnified views of spherical balls at ends.

The stents were shape set from NiTi wire using a template as shown in Fig. 5(a) by heating to 520 $^\circ$C for 30 minutes followed by immediate quench in room temperature water. To minimize airway trauma and to assist in delivery and removal, the ends of the helix are spherical balls formed by melting the wire with a TIG welder, Fig. 5(b).

### B. Phantom Trachea Model

**Fig. 6.**
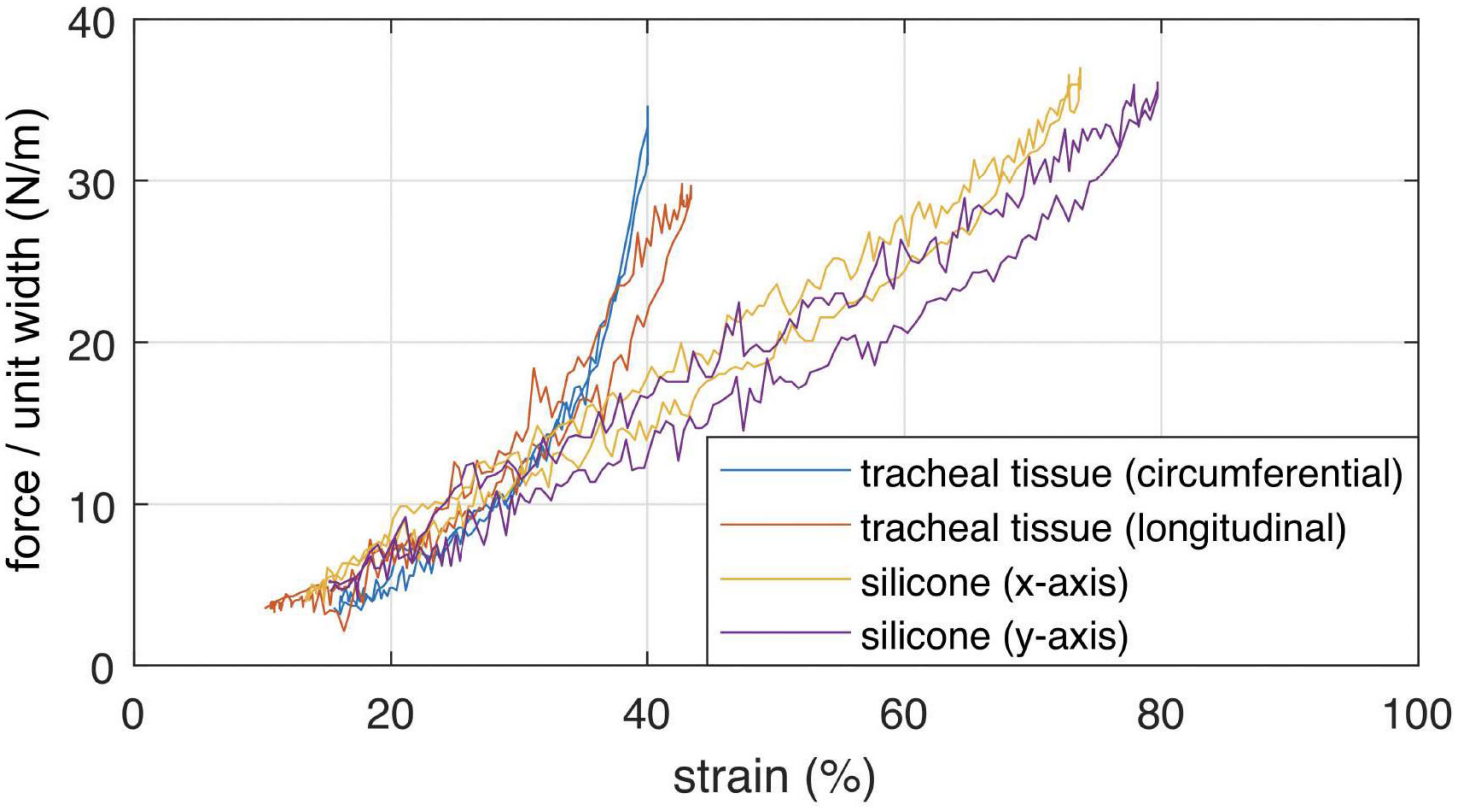
Force per unit width versus strain curves for membranous porcine tracheal tissue and phantom silicone. The $x$ and $y$ axes refer to arbitrary orthogonal directions on the planar silicone sample.

A phantom trachea model was developed owing to the difficulty of creating a consistent ex vivo tracheobronchomalacia model in which the cartilage is removed while leaving the remaining tracheal tissue intact. To approximate actual tracheal tissue properties, we collected samples of fresh porcine tissue from the membranous portion of the trachea (from the gaps between the cartilage rings). We then measured the tissue stiffness on a biaxial tester and iteratively arrived at a silicone phantom that matched the initial tissue stiffness, Fig. 6. The resulting tracheal phantom was 0.26 mm thick (TrueSkin 10, Quantum Silicones). Silicone exhibits a large linear stress-strain region compared to the stiffening characteristic of tracheal tissue. Consequently, area reduction of the silicone trachea model will overestimate that of tissue.

### C. Experimental Procedure

**Fig. 7.**
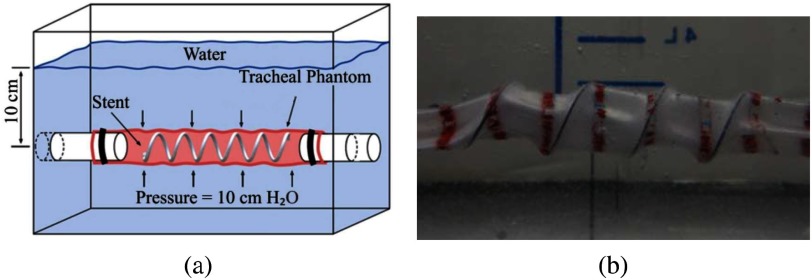
Phantom trachea pressure experiments. (a) Schematic of tank test. (b) Stented silicone trachea under 10 cm H$_2$O pressure.

The phantom trachea was mounted on two co-axial rigid plastic tubes that extended through the walls of a tank as shown in Fig. 7(a). The two tubes enabled access to the inside of the phantom from both ends. Mineral oil was distributed on the inner surface of the phantom to reduce friction between the silicone trachea and the stent and so mimic the slippery endothelial surface of the trachea. To deliver a stent into the phantom, sutures were tied to each end of the stent so that it could be stretched and so reduced to a diameter less than that of the phantom trachea. One suture was then passed through the phantom and the stent was then positioned in the middle of the phantom. The phantom was then pressurized to increase its diameter and so allow the stent to assume a relaxed configuration. After removing the pressure, the tank was then filled with deionized water to a depth of 10 cm from the central axis of the phantom trachea.

**Fig. 8.**
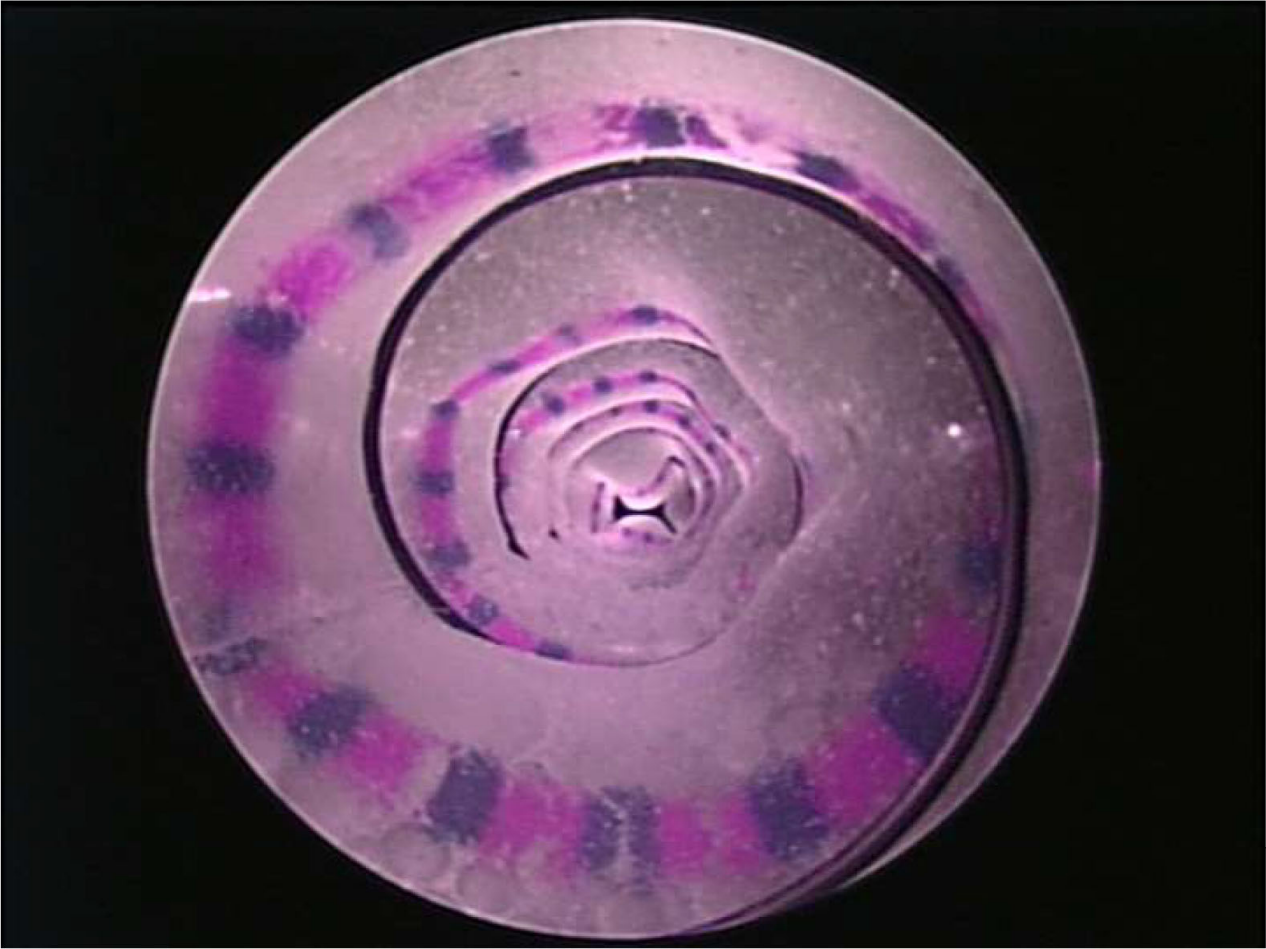
Measurement of phantom cross-sectional area using telescope images.

To measure the tracheal phantom cross sectional area, red circumferential curves were created every 1cm along the length (Fig. 7) with blue marks spaced 2.5 mm apart on the red lines (Fig. 8). By inserting a telescope (Karl Storz 10328AA) inside the artificial trachea, the cross section was measured as the area inside a red curve using the blue marks to calculate distance at the image depth of any given red curve. The camera calibration toolbox for Matlab [28] was used to estimate camera parameters and to correct for image distortion. Calibration using closed curves of known area was performed yielding mean and maximum errors of 0.9% and 2.6%, respectively.

To measure the reduction in stent diameter and cross sectional area as modeled in Section II, images were taken from the top of the tank using a camera (Canon EOS 6D Mark II) with a macro lens (Canon EF 100 mm). A 5 mm pitch checkered pattern was imaged and Matlab's camera calibration toolbox was utilized to estimate the camera parameters for correcting image distortion due to refraction through water. Calibration yielded a maximum error of 0.9 pixels or 0.04 mm at 51 pixels/mm. Using the calibrated images, lines were fit to the outer edges of the two central coils and the minimum distance between the coil edges and the opposing line was taken as the reduced stent diameter.

**Fig. 9.**
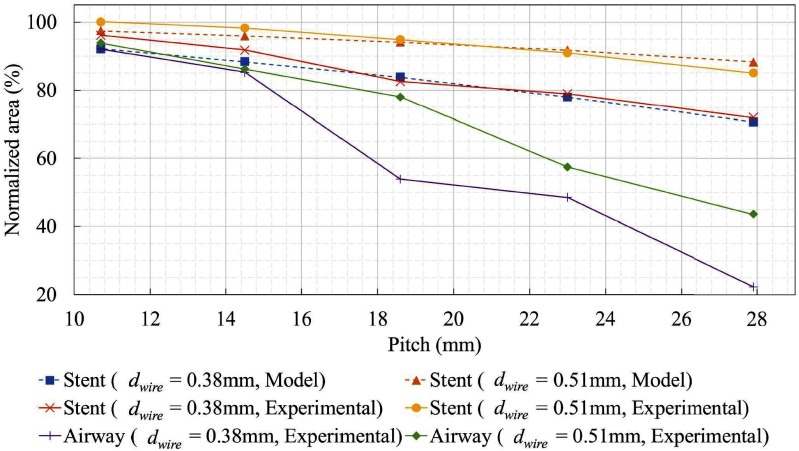
Stent and phantom trachea airway area reduction versus stent pitch for two wire diameters at 10 cm H$_2$O. Dashed curves show area reduction predicted by model of Section II-A.

A comparison of experimentally-measured and model-predicted cross-sectional areas is plotted in Fig. 9 as a function of stent pitch. The areas are normalized with respect to the unpressurized cross-sectional area. The effect of external pressure on the stent is to cause it to lengthen and reduce in diameter and cross-sectional area. The model closely predicts the experimentally observed reduction in stent cross sectional area ($<\text{ 5}\%$).

While stent cross-sectional area decreases with increasing stent pitch, the major effect of increasing pitch is the reduction in cross section associated with tracheal phantom stretching between the coils (as illustrated in Fig. 2(b)). These results suggest that a stent pitch of 14.5 mm will reduce tracheal cross section area by 15% for either wire diameter. Since a real malacic airway will have more support than the phantom employed here, larger pitches can likely be employed.

## IV. Stent Delivery and Removal Tools

**Fig. 10.**
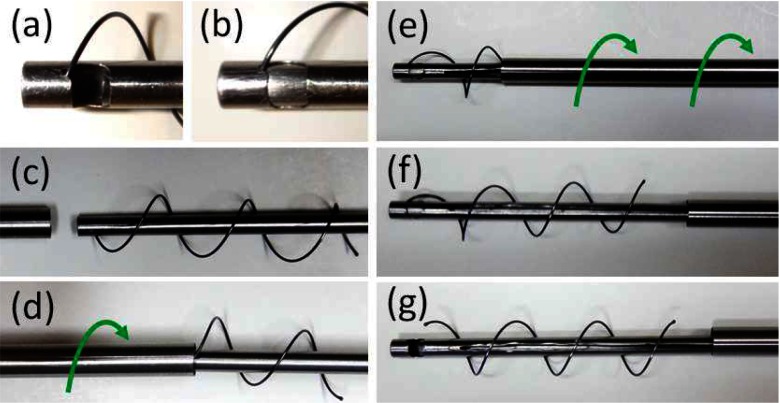
Stent delivery tool. (a) and (b) Prior to delivery, ball on distal end of stent is locked between the inner two cannulas. (c) and (d) Stent is then compressed and housed inside the outer cannula using a screwing motion. (e) Once distal end of the instrument is placed at desired location in trachea, outer cannula is retracted using an unscrewing motion. (f) and (g) Retraction of the innermost cannula releases the ball on the distal end of the stent.

To enable atraumatic stent delivery and removal, bronchoscope-guided instruments were developed. The stent delivery system is comprised of three concentric cannulas. The stent is preloaded into the delivery system and subsequently deployed as shown in Fig. 10. The cannulas are sized such that a flexible bronchoscope (Karl Storz 10328AA) fits inside the lumen of the innermost cannula enabling image-based positioning in the trachea.

**Fig. 11.**
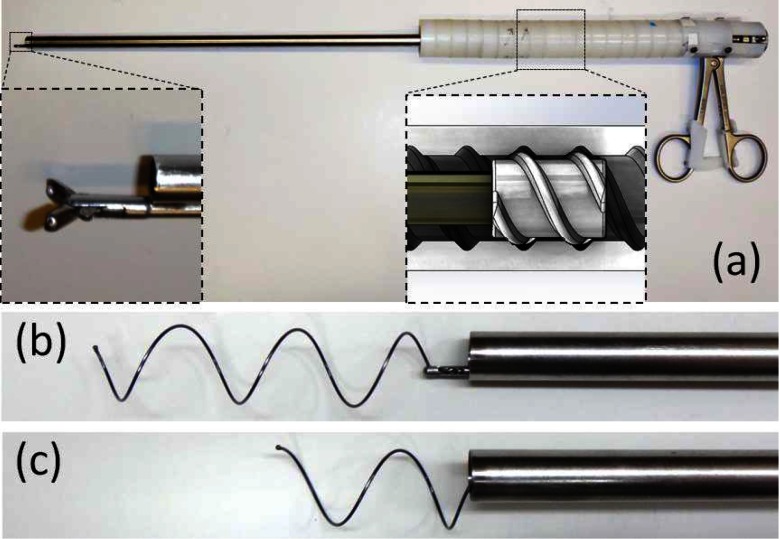
Stent removal tool. (a) Magnified view (bottom left corner) shows grasping forceps. Diagram (bottom right) shows screw threads of helical joint. Outer white threads are attached to outer cannula. Inner gray threads are attached to grasping forceps. (b) Tool grasping stent. (c) Stent is shown partially “unscrewed” from the trachea into cannula.

To achieve atraumatic stent removal even when the stent has been endothelialized, the removal system was designed to retract the stent using an unscrewing motion matching the helical pitch of the stent. The system is comprised of optical forceps (through which a bronchoscope can be inserted) that fit inside an outer cannula, Fig. 11. The two components are connected at their proximal ends by a helical joint matching the pitch of the stent.

For stent removal, the forceps are first used to grasp the ball on the proximal end of the stent. Holding the outer cannula fixed in place, the forceps handle is then rotated. The helical joint causes the inner cannula to follow a helical retraction path causing the stent to be retracted in a follow-the-leader fashion into the outer cannula.

**Fig. 12.**
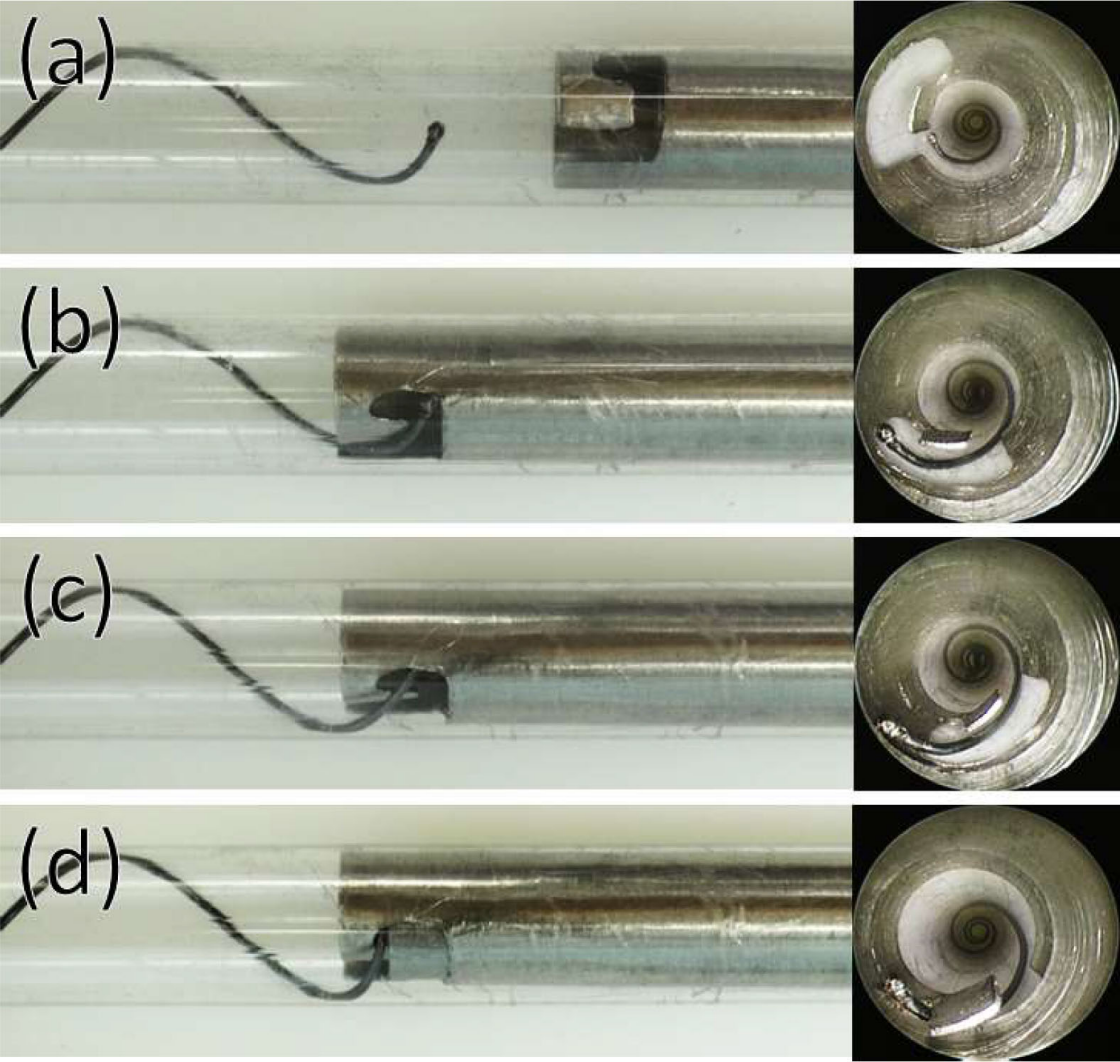
Regrasping partially retracted stent. Device is shown inside clear plastic cannula for visualization. Bronchoscopic image used during regrasping is also shown. (a) Tool is extended so that stent ball enters tool slot. (b) and (c) Tool is rotated to position stent ball inside notch of tool. (d) Inner cannula is used to grasp stent and lock ball in place.

During retraction into the outer cannula, the stent ball would occasionally slip out of the optical forceps. Since the stent ball was then pressed against the inside of the outer cannula, it was difficult to regrasp with the forceps. For these situations, the regrasping device of Fig. 12 was used. The device is comprised of a notched outer cannula and cylindrical inner cannula. The notched outer cannula functions like a spatula. Under bronchoscopic guidance, the notched cannula is extended such that the ball enters the notch. Rotation of the notched cannula causes the ball to slide onto the interior of this cannula from where it can be positioned at the innermost corner of the notch and locked in place using the inner cannula. Once regrasped, helical stent retraction can then proceed as described above.

## V. In Vivo Experiment

To evaluate stent safety as well as the delivery and removal tools, a 28-day in vivo experiment was conducted on a 20 kg (2 month old) Yorkshire swine. The stent was positioned approximately 2 cm proximal to the carina on the first day. It was removed on the 21st day and the animal was survived for an additional 7 days. Weekly bronchoscopy examinations and chest x-rays were performed. All procedures were approved by the Institutional Animal Care and Use Committee.

### A. Stent Design

Based on the phantom study results of Fig. 9, a stent with a pitch of 18.6 mm and a wire diameter of 0.51 mm experiences a cross-sectional area reduced to 77% of the nominal area under a pressure of 10 cm H$_2$O. Since the tracheal phantom was completely unsupported, this value represents a worst-case area reduction. Consequently, these parameters would likely provide sufficient airway protection and so were selected for the in vivo test. The 18.6 mm pitch of the phantom studies corresponds to a helix angle of 25$^\circ$ so this helix angle together with a wire diameter of 0.51 mm were used to design stents for the in vivo experiment.

Since the tracheal diameter of the pig was not known in advance, stents were prepared in 5 diameters (10.03, 11.62, 13.21, 14.80 and 16.38 mm) and two lengths, corresponding to 2 and 3 complete coils, respectively. At the start of the procedure, a chest x-ray was taken and the inner diameter of the trachea was estimated to vary between 11.4 mm to 13.2 mm in the desired region of stent deployment. To avoid stent migration, the stent should be preloaded against the tracheal tissue and so a diameter of 13.21 mm was selected for deployment. In addition, stent length was selected to be 3 coils so that approximately half of the trachea was stented.

### B. Stent Deployment and Performance

**Fig. 13.**
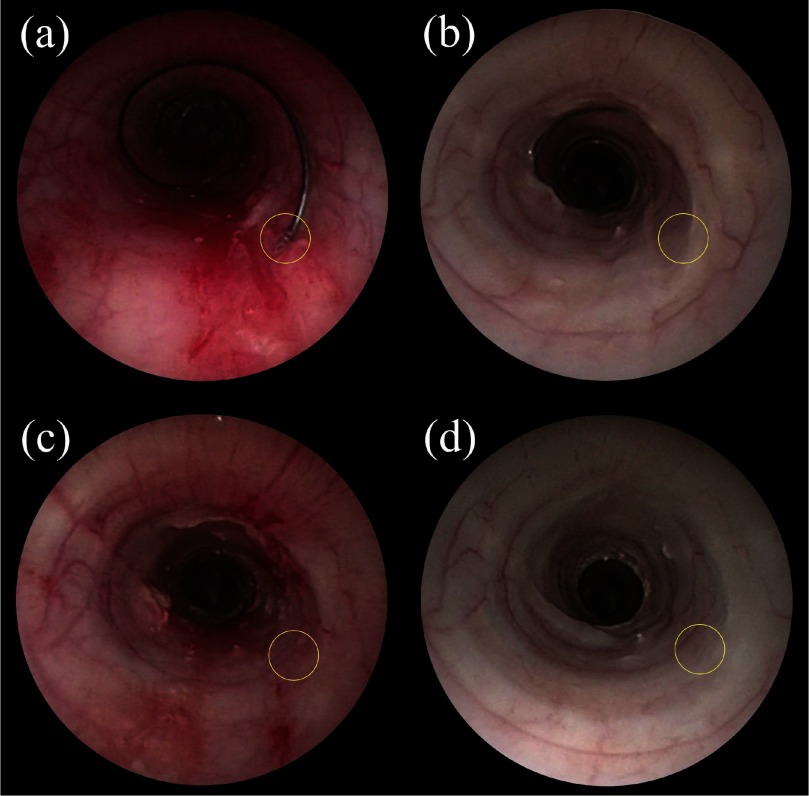
Bronchoscopic images of the trachea. (a) Immediately after stent deployment. (b) 21 days after stent deployment. (c) Immediately after stent removal. (d) One week after stent removal. The location of the ball on the proximal end of the stent ball is circled.

Stent deployment took 10 min 15 sec and the deployed stent is shown in Fig. 13(a). The stent was well tolerated by the animal. No coughing or other respiratory distress was observed at any time during the course of the experiement. Bronchoscopic imaging showed that, while some mucus collected on the stent wire, the tracheal tissue between the helical coils was free of mucus.

**Fig. 14.**
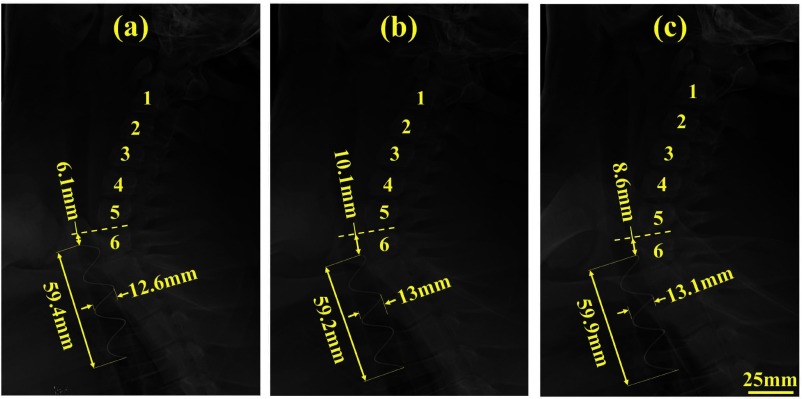
Lateral view x-ray images taken on (a) day of stent deployment, (b) day 7 after stent deployment, and (c) day 21 after stent deployment. Numbers 1–6 indicate vertebrae. Measurements indicate displacement with respect to 6th vertebra as well as diameter and length of stent.

To investigate stent migration, x-ray images were taken at the time of stent placement and then at 7, 14 and 21 days. From these images, stent length, diameter and position with respect to the vertebrae were measured (Fig. 14). Stent position along the vertebrae was observed to vary by up to 4mm. This apparent displacement, however, may be due to differences in positioning the animal during x-ray since bronchoscopic examination showed that the stent became partially embedded in the trachea over the course of the experiment (Fig. 13(b)).

### C. Stent Removal and Tracheal Recovery

Stent removal took 14 min 19 sec. As shown in Fig. 13(b), the ball on the proximal end of the stent was embedded in the tracheal epithelium. After suctioning the trachea to remove mucus, the removal instrument was inserted and the forceps were used to excise the ball from the tissue. Several attempts were required to securely grasp the ball. Once grasped, the stent was gently removed by “unscrewing” it from the trachea and into the outer cannula of the removal tool.

Fig. 13(c) depicts the trachea just after stent removal. The location where the ball was embedded in the tissue is visible, but the tracheal epithelium is relatively undamaged. Fig. 13(d) shows the same region of the trachea one week after stent removal. The tissue has healed and possesses normal coloration. While a small amount of mucus is visible along portions of the helical footprint of the stent, the airway is not at all obstructed.

## VI. Conclusion

There is a critical need for airway stents to improve the treatment of children with tracheobronchomalacia. The helical stents proposed here hold promise in addressing this need. To design these stents, a mechanics-based model was derived for computing the change in stent diameter associated with an applied external pressure. To understand and model tracheal contraction in the unsupported regions between the stent coils, a phantom model was constructed based on porcine tracheal tissue properties. This model provides an estimate of worst-case tracheal area reduction in response to an external pressure.

This model was used to design a stent along with delivery and removal tools that were tested in an in vivo porcine model. The in vivo experiment confirmed that the proposed helical stent meets the design goals: It resists migration along the airway, allows mucus flow and can be removed with minimal trauma. Furthermore, the stent proved to be safe and was well tolerated by the animal. Additional studies are needed to further develop the stent and tools and to perform a more detailed performance assessment.
